# Secretome Analysis for a New Strain of the Blackleg Fungus *Plenodomus lingam* Reveals Candidate Proteins for Effectors and Virulence Factors

**DOI:** 10.3390/jof9070740

**Published:** 2023-07-11

**Authors:** Nahla A. Bouqellah, Nadia A. Elkady, Peter F. Farag

**Affiliations:** 1 Department of Biology, College of Science, Taibah University, P.O. Box 344, Al Madinah Al Munawwarah 42317-8599, Saudi Arabia; 2 Department of Microbiology, Faculty of Science, Ain Shams University, Cairo 11566, Egypt; nadiaelkady@sci.asu.edu.eg (N.A.E.); peter_jireo@sci.asu.edu.eg (P.F.F.)

**Keywords:** computational annotation, network analysis, pathogenic fungi, proteomics

## Abstract

The fungal secretome is the main interface for interactions between the pathogen and its host. It includes the most important virulence factors and effector proteins. We integrated different bioinformatic approaches and used the newly drafted genome data of *P. lingam* isolate CAN1 (blackleg of rapeseed fungus) to predict the secretion of 217 proteins, including many cell-wall-degrading enzymes. All secretory proteins were identified; 85 were classified as CAZyme families and 25 were classified as protease families. Moreover, 49 putative effectors were predicted and identified, where 39 of them possessed at least one conserved domain. Some pectin-degrading enzymes were noticeable as a clustering group according to STRING web analysis. The secretome of *P. lingam* CAN1 was compared to the other two blackleg fungal species (*P. lingam* JN3 and *P. biglobosus* CA1) secretomes and their CAZymes and effectors were identified. Orthologue analysis found that *P. lingam* CAN1 shared 14 CAZy effectors with other related species. The Pathogen-Host Interaction database (PHI base) classified the effector proteins in several categories where most proteins were assigned as reduced virulence and two of them termed as hypervirulence. Nowadays, in silico approaches can solve many ambiguous issues about the mechanism of pathogenicity between fungi and plant host with well-designed bioinformatics tools.

## 1. Introduction

Blackleg, also known as phoma stem canker, is a destructive disease of *Brassica* crops (canola, oilseed rape) that causes about USD 1 billion in global crop losses every year [1,2,3]. *Plenodomus lingam* (currently named as *Leptosphaeria maculans*, https://www.indexfungorum.org/names/NamesRecord.asp?RecordID=416165 accessed on 4 April 2023) is the main causal agent of blackleg disease, followed by *Plenodomus biglobosus* (Syn. *Leptosphaeria biglobosa*), a comparatively less aggressive species due to its inability to secrete phytotoxin sirodesmin PL [4,5,6]. *P. lingam* is a hemibiotrophic pathogen that can survive on crop residues from season to season after the harvest, providing favorable conditions for the development and maturation of pycnidia and pseudothecia. Pycnidiospores and ascospores are released from their fruiting bodies as primary inocula for infection [7,8]. In addition, this fungus can attack and infect all parts of the plant, causing up to 50% yield losses in individual fields of different countries, including European countries, Canada, and Australia [2,9].

Fungi secrete a wide range of proteins outside the plasma membrane of the cell, defined as fungal secretome, which play a pivotal role in decaying their substrates [10,11] and interacting with their plant hosts [12,13,14,15]. The fungal secretome includes lipases, proteases, Carbohydrate-Active enZymes (CAZymes), proteins of unknown function (hypothetical proteins), and Small Secreted Proteins (SSP) [16]. Most secretomes carry a signal peptide (SP), a signal sequence at the N-terminus that contains 3 parts: a positively charged n-region, a polar c-region, and a hydrophobic region that forms an alpha helix (h-region) between the n- and c-regions [17]. This signal peptide leads to translocation of proteins through the endoplasmic reticulum and the Golgi compartment, allowing them to reach the extracellular space [18,19]. The important function of extracellular proteins is to interact with the environment of the fungus for breaking down the needed nutrients and determining its virulence against the host plant [20].

Effector proteins are the most important class of proteins for the interaction between the pathogen and host [21]. Their secretions are either inside the host cell (cytoplasmic effectors) or outside the host cell (apoplastic effectors), where they manipulate the metabolic processes inside the host and facilitate the effector-triggered susceptibility (ETS) [22,23]. Most effectors that interact with the plant immune system are either virulence (Vir) proteins or avirulence (Avr) proteins. The successful penetration and colonization of the pathogen into the host cell relies on overcoming the multiple layers of plant immunity [24]. The first layer of plant immunity is triggered by a pathogen-associated molecular pattern (PAMP) that recognizes the pathogen molecules by specific membrane-localized receptors, leading to a defence response called PAMP-triggered immunity (PTI) [25]. Although PTI is effective against different microorganisms, pathogens defeat it by effectors. The second layer of plant immunity is triggered by another type of receptor proteins called resistance (R) proteins [26]. Avirulence proteins are the type of effectors that are recognized by these specific R proteins in the host to trigger a strong defence response called hypersensitive response (HR), a programmed cell death at the site of infection [27,28,29].

Currently, few previous data on *Plenodomus lingam* secretome are available. Therefore, our knowledge of important secreted proteins of *P. lingam* and their functions is still ambiguous. Until 2021, only three recorded strains of *P. lingam* were available on the NCBI genome database (https://www.ncbi.nlm.nih.gov/genome/browse/#!/eukaryotes/11473/, accessed on 5 April 2023). The codon sequence (CDS) was described for only one strain (*P. lingam* JN3, formerly *Leptosphaeria maculans* JN3) [30]. Several proteins of this strain were characterized on the UniprotKb database (https://www.uniprot.org/uniprotkb?query=leptosphaeria%20maculans%20JN3, accessed on 6 April 2023). In 2022, new data appeared on the NCBI database for a new strain named *P. lingam* CAN1, which was chosen for this study. Its proteome was not previously characterized or annotated. We used the sequenced draft genome of *P. lingam* CAN1 for an in silico prediction and annotation of its secretome. This allowed the characterization and identification of the possible function of most proteins, to understand their role in pathogenesis. Also, a comparative analysis between *P. lingam* CAN1 and two other related strains (*P. lingam* JN3 and P. *biglobosus* CA1) was described especially for CAZymes and effector proteins to detect evolutionary relationships. Eventually, we predicted effector candidates to define a set of sequences that will serve as a starting point for further studies on the pathogenicity mechanisms of *Plenodomus* species.

## 2. Materials and Methods

### 2.1. Sequence Information and Retrieval

The information data of *Plenodomus lingam* strain CAN1 (accession no. JACTNS010000001.1) was released on NCBI database by the Chinese Academy of Inspection and Quarantine in February 2022 (https://www.ncbi.nlm.nih.gov/genome/11473?genome_assembly_id=1795175, accessed on 5 April 2023). The genome of this strain has a length of 42,037,800 bp and contains 11,989 genes encoding 11,837 proteins. Out of these, 11,098 (93.75%) of the proteins were classified as hypothetical while 739 (6.25%) were fully characterized proteins. The proteome of *P. lingam* CAN1 was retrieved from the NCBI database (https://www.ncbi.nlm.nih.gov/gnome/browse/#!/prteins/11473/1795175%7CPlenodomus%20lingam/Un/, accessed on 5 April 2023). Moreover, the retrieved protein sequences were further explored in UniProt to determine the availability of annotation data using a batch search (accessed on 5 April 2023).

### 2.2. Prediction of Secretome

The secretome was predicted in a similar manner to the guidelines of the Fungal Secretome KnowledgeBase (FunSecKB) [31] and a previously described pipeline [32]. These pipelines predicted the secreted proteins from the sequences that carry a signal peptide without any transmembrane helix domain and glycophosphatidylinositol anchor motifs (GPI). For the prediction of the signal peptide, the first filtration and screening were performed by SignalP (version 5.0) [33]. DeepTMHMM V1.0.24 servers were used to remove the transmembrane helix proteins [34,35]. The endoplasmic-reticulum-targeting protein sequences were excluded by the Prosite database with the ScanProsite web server [36]. The proteins harboring GPI motifs were predicted using NetGPI (version 1.1) [37].

### 2.3. Characterization and Annotation of Secretory Proteins

For the prediction of protein families and GO (gene ontology) terms of the refined secretome, InterPro V93.0 [38] and orthologous matrix browser (Oma browser) [39] were used. KOBAS 3.0 [40] was used for the KEGG (Kyoto Encyclopedia of Genes and Genomes) pathway enrichment analysis using the hypergeometric test/Fisher’s exact test as a statistical method and Benjamini and Yekutieli as FDR correction methods, and *p* < 0.05 was set as the cut-off criterion. The physicochemical properties of proteins were characterized by the ProtParam tool [41]. The output properties from this sever include molecular weight (MW), theoretical isoelectric point (PI), extinction coefficients (EC), instability index (II), aliphatic index (AI), and grand average of hydropathicity (GRAVY). For a detailed annotation of carbohydrate-degrading enzymes, the CAZy database and dbCAN3 server HMMER (E-Value < 1 × 10^−15^, coverage > 0.35) and DIAMOND (E-Value < 1 × 10^−102^) were used [42,43]. Proteolytic enzymes were annotated using a BlastP search against MEROPS database release 12.4 [44], while lipases were identified using the Lipase Engineering Database (LED) V4.1.0 (E-Value cutoff 1 × 10^−5^) [45]. For the effector prediction, EffectorP 3.0 was used [46].

### 2.4. Analysis of the Putative Effectors

The predicted effector candidates were scanned against the Pathogen–Host Interaction database (PHI-base) for exploring the virulence factors [47]. STRING V11.5 was used to predict protein–protein interaction (PPI) networks, providing functional associations between proteins to classify the effectors into groups and helping in sieving the dominant effectors in the pathogenicity process [48]. The KAH9875744.1 protein was selected as a case study for more confirmatory analysis due to (i) high probability values for both apoplastic and cytoplasmic effectors, (ii) involving the dominant CAZyme effector groups according to the STRING web server, and (iii) its unique evolutionary features (outgroup branch) compared with other related clustering proteins. The 3D structure of the candidate protein was done with the SWISS-MODEL server [49] based on homology modeling. Docking analysis was performed using CB-Dock2 software [50] from the downloaded 3D structure model. It helps the study and prediction of how ligands (pectin, pectic acid, and digalacturonate) interact with protein (pectate lyase). In addition, a BLASTp search from NCBI against the non-redundant database with default parameters was run to find the homologues of the protein. Alignment and a phylogenetic tree were constructed using the Multiple Sequence Comparison by Log-Expectation (MUSCLE) algorithm and Clustal Omega [51]. The phylogenetic tree was displayed and visualized via iTOl V6 [52]. Signature motifs within the sets of similar proteins were detected using the Multiple Expectation-maximization for Motif Elicitation (MEME suite) V5.5.2 with a maximum number of 15 motifs, a range of 2–10 sites per motif, and an E-value of less than 0.05 [53].

### 2.5. Comparative Analysis of Effector and CAZymes among Species and Isolates

The secretome, putative effector proteins, and CAZymes of *P. lingam* CAN1 were compared with another two blackleg-related strains (*P. lingam* JN3 and *P. biglobosus* CA1). The same workflow was applied using the above-mentioned bioinformatic software for the fungus *P. lingam* CAN1. Also, whole-genome comparison and orthologous clustering annotation between the three strains were done with the OrthoVenn3 web server with e-value cut-off 1 × 10^−5^ [54].

## 3. Results and Discussion

Agricultural crops supply over 80% of the food consumed by humans and are an important source of nutrition for livestock. Unfortunately, plant diseases often threaten the availability of plants for human and animal consumption [55,56]. Fungi are the main microbial pathogens that cause huge yield losses in agricultural crops and post-harvest products, thus threatening food security all over the world [57]. As a result, the global economy loses about $220 billion annually due to fungal diseases [55]. To prevent such fungal invasions against plants, a deep understanding of the effector proteins secreted by the fungal pathogens and the plant immune response is necessary to achieve more durable resistance against the pathogens [58]. In silico analysis of the secretome is a powerful tool to aid in the management of fungal infection in plants [59].

### 3.1. Prediction of Secretome

**Figure 1:**
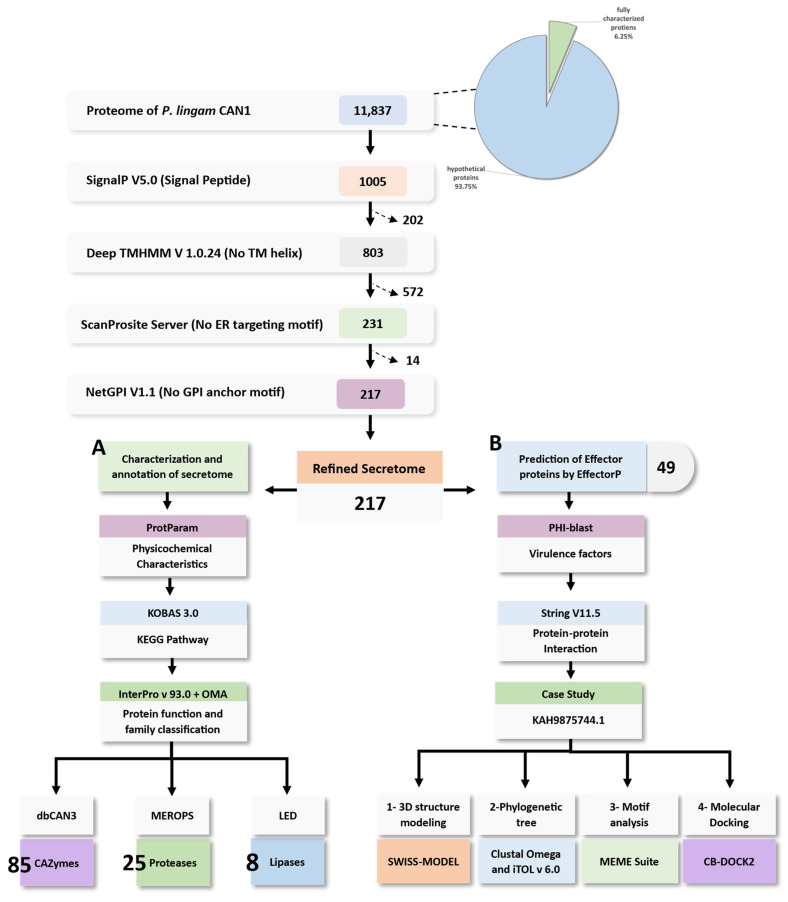
The pipeline used for the prediction of *P. lingam* CAN1 secretome and characterization of different enzyme families and putative effectors: (**A**) Characterization and annotation of secretome pathway; (**B**) Prediction and characterization of effector proteins pathway.

Our workflow starts with the complete proteome of *P. lingam* strain CAN1 (accession no. JACTNS010000001.1) extracted from NCBI (Table S1). From a total of 11,098 hypothetical proteins, 1005 signal peptides were predicted using SignalP V5.0. The prediction was then refined using DeepTMHMM V1.0.24 servers to remove 202 proteins harboring transmembrane helix proteins. A total of 572 proteins containing endoplasmic-reticulum-targeting protein sequences were excluded by the ScanProsite web server. Fourteen proteins were then identified by NetGPI to harbor GPI motifs, resulting finally in a list of 217 refined secreted proteins (Figure 1).

### 3.2. Characterization of Physicochemical Properties of Secretory Proteins

**Figure 2:**
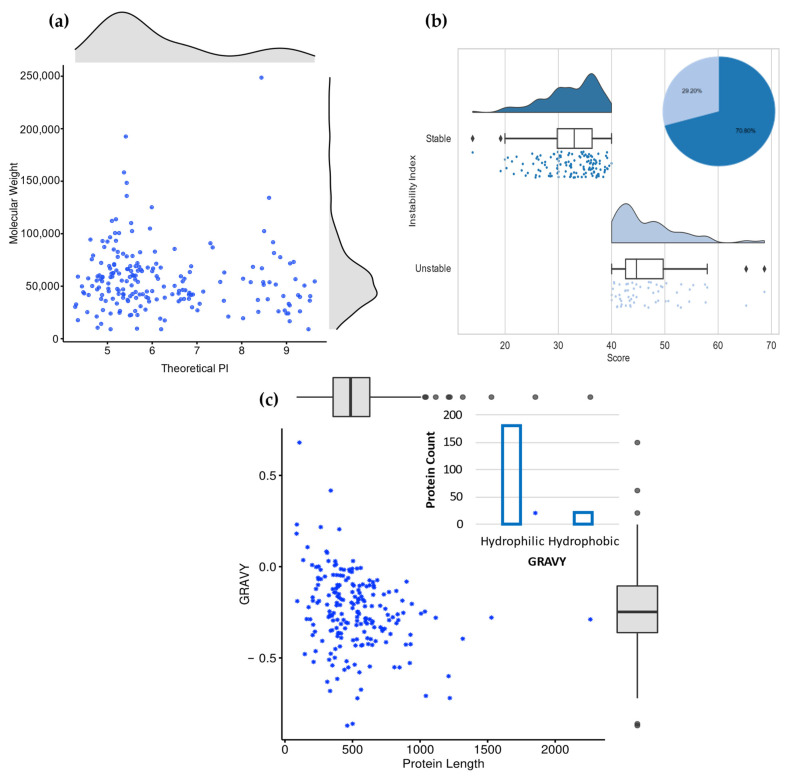
Physicochemical characteristics of the secretome using ProtParam tool: (**a**) Isoelectric point vs. molecular weight properties of the refined secretome; (**b**) Instability index property that classifies stable proteins (blue box densities) and unstable proteins (faint blue box densities); (**c**) Protein length vs. GRAVY scores, where positive values were categorized as hydrophobic (membrane) proteins, while negative values were categorized as hydrophilic (globular) proteins.

The work pipeline on the refined secretome proceeded in two directions. The first was the characterization and annotation of the 217 secreted proteins. The second was exploring and identifying effector proteins and virulence factors. Physiochemical parameters in proteins can define their behavior and stability under different in vitro conditions [60]. The physicochemical properties of the secreted proteins, including molecular weight (MW), theoretical isoelectric point (PI), extinction coefficients (EC), instability index (II), aliphatic index (AI), and grand average of hydropathicity (GRAVY) were computed using the Expasy ProtParam tool (Table S2, Figure 2). The maximum observed molecular weight was 248,642.97 Daltons (da), whereas the minimum molecular weight observed was 9042.34 da. For the theoretical PI values, only 45 proteins were basic (7.01–9.63), while 172 proteins were acidic (4.28–6.91). The PI of any protein is the pH at which the net charge carried by the molecule surface is zero (Figure 2a) [61]. It determines the stability, solubility, and activity of a protein and its interactions with other molecules in different pH environments [62]. Only 60 (27.6%) proteins of the refined secretome were considered unstable according to their instability indices. Cut-off values of <40 and >40 were used to identify the stable and unstable proteins (Figure 2b). The instability index estimates the stability of a protein in vitro; a stability index of less than 40 is considered stable [61].

The values of computed EC ranged from 2115 to 265,185 M^−1^ cm^−1^. EC is a measure of how much light a mole of protein absorbs at a specific wavelength, most commonly 280 nm. It reflects the concentration of tryptophan, cysteine, and tyrosine in that protein, where high EC values indicate the presence of high concentrations of these amino acids (Table S2). EC allows studying protein–ligand and protein–protein interactions [61,63]. Proteins rich in cysteine residues display high stability against high temperature, proteolysis, and pH changes and are predicted to play an important role during host–pathogen interactions [64]. The AI refers to the relative volume occupied by a protein’s aliphatic hydrophobic side chains of amino acids such as V (valine), A (alanine), L (leucine), and I (isoleucine). It is an indicator of the thermal stability of proteins: the higher the AI value, the more stable the protein is at high temperatures [63,65]. AI values of the studied secretome reflected high thermostability of 177 proteins (70.09–95.97) over wide temperature ranges (Table S2).

Another important parameter studied was the GRAVY score. It determines the hydrophobic or hydrophilic nature of proteins [63]. The GRAVY score is the sum of hydropathy values of all amino acids present in the protein, divided by the number of residues in the same protein. Negative gravy score values indicate hydrophilicity, while positive values indicate hydrophobicity [63]. The GRAVY score of 200 proteins was found to be negative, with values ranging from −0.002 to 0.721 (Figure 2c). The negative GRAVY score predicts that these proteins could be hydrophilic (polar) with good solubility, rather than hydrophobic (non-polar) [65].

### 3.3. Characterization and Annotation of Secretory Proteins

**Figure 3:**
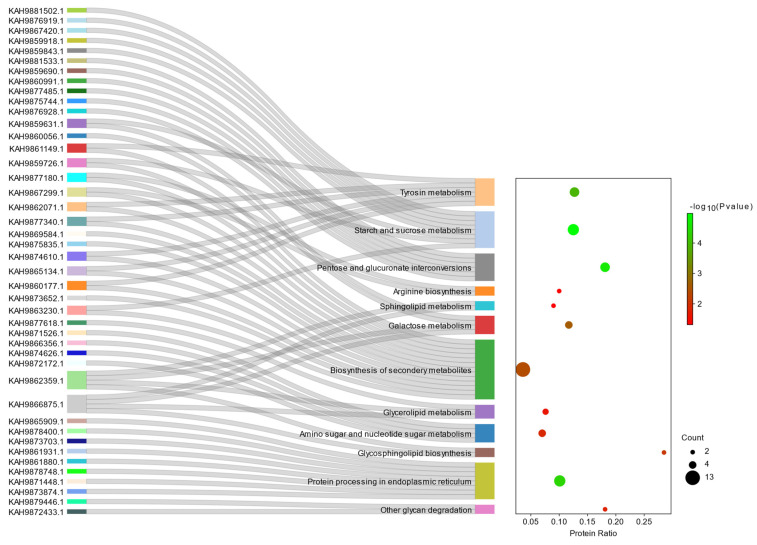
Statistics of KEGG enrichment pathway (*p* < 0.05) of the refined secretome where the minimum value of the –log10 (*p*-value) scale is 1 and the maximum value of the –log10 (*p*-value) is 5.

KEGG pathway enrichment analysis was used for functional annotation of the predicted secretome by KOBAS 3.0, a bioinformatic tool that consists of two parts: the annotation module and the enrichment module [66]. The annotation module accepts a protein list as input and annotates each protein based on multiple databases of known pathways. The enrichment module gives an answer about which pathways and GO terms are statistically significantly associated with the input list [66,67]. Enriched functional terms associated with KEGG pathways are summarized in Figure 3. KEGG analysis categorized 43 secreted proteins into 12 pathways (*p* < 0.05). Among all pathways, biosynthesis of secondary metabolites, starch and sucrose metabolism, protein processing in the endoplasmic reticulum, and tyrosine metabolism were the top four pathways. Secondary metabolites have been shown to play an important role in the pathogenicity of several fungal pathogens. They are beneficial for the infection process and contribute to adjusting the disease progress [68]. They act in multiple ways and increase the pathogen’s ability to overcome unfavorable conditions in their host environment in addition to tolerance to several stressful environmental factors, including heat, drought, and UV light [69]. Moreover, starch and sucrose metabolism are responsible for host starvation by direct depletion of the host’s carbon reserves and increased carbon consumption, as mentioned in previous studies [70].

**Figure 4:**
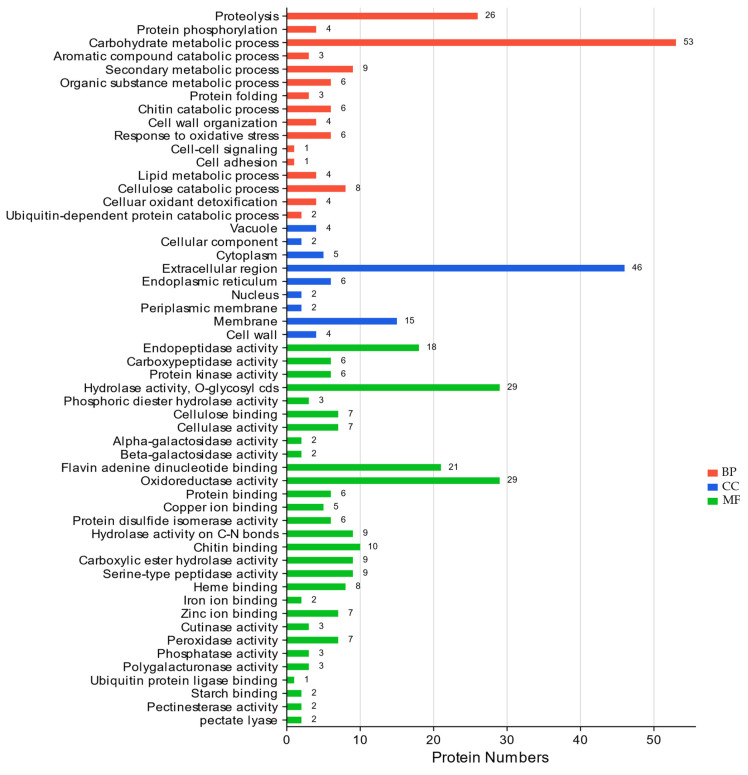
Gene ontology (GO) terms: BP (Biological Process); CC (Cellular Component); MF (Molecular Function) for the predicted secretome describing the protein functions.

The results also showed that the predicted secretome was functionally annotated in many biological processes, cellular components, and molecular functions. We filtered 54 GO terms at each time point in which the secretome was described (Figure 4). The most annotated GO terms in the biological processes were the carbohydrate metabolic process (GO:0005975), and proteolysis (GO:0006508), while the extracellular region (GO:0005615) and membrane (GO:0005886) were the most encountered GO terms in the cellular component. Hydrolase activity, O-glycosyl compounds (GO:0004553), flavin adenine dinucleotide binding (GO:0071949), and oxidoreductase activity (GO:0016491) were the most enriched terms in the molecular functions. Being a plant pathogen, it is not surprising that the secretome of *P. lingam* CAN1 consists mainly of proteins responsible for carbohydrate metabolic process and in catabolic processes. Pathogens usually utilize their plant hosts as a source of nutrients, and so carbohydrate-degrading and catabolic enzymes are crucial [71].

**Figure 5:**
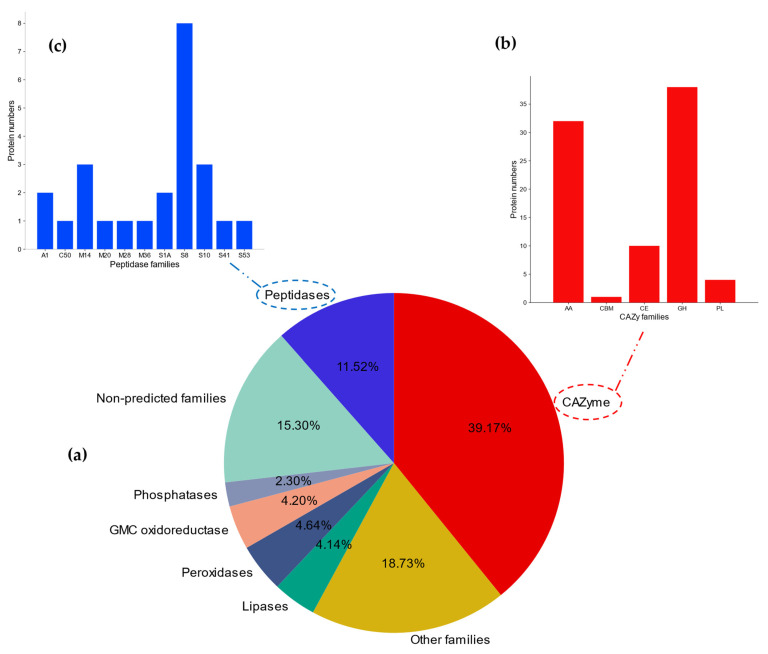
Classification of enzyme families: (**a**) Pie chart of total enzyme families for the refined secretome; (**b**) Different family groups of CAZymes; (**c**) Different family groups of peptidases.

Out of the 217 proteins of the predicted secretome, 85 were identified as carbohydrate-active enzymes, representing 39.17% of the secretome (Figure 5a,b). Peptidases constituted approximately 11.52%, with a total of 25 enzymes (Figure 5a–c). Eight lipases were recorded, representing 4.14% of the secretome. Oxidoreductases, peroxidases, and phosphatases represented 11.14% of all the enzymes combined (Figure 5a). Plant cell-wall-degrading enzymes play significant roles in fungal pathogenicity [71], allowing them to attack and penetrate their host’s cell wall. Cellobiose-related enzymes included 10 gluco-oligosaccharide oxidase and 7 GMC oxidoreductases, belonging to families AA7 and AA3, respectively, were the most identified CAZy proteins (Table 1).

Five multicopper oxidases (AA1) and 4 lignin peroxidases (AA2) were also recorded. These enzymes can exhibit important auxiliary roles in lignocellulose degradation [72]. Five galactanase enzymes (GH16) were also identified which are important for hydrolysis of galactan (components of many plant cell walls) [73]. Many secreted peptidases are described as virulence factors in fungal pathogens. They can suppress their host’s defense responses through inactivation or modification of the host defense proteins [74]. In our study, the most frequently identified peptidases were subtilisin-related (8 out of 25), belonging to family peptidase S8 (Figure 5a–c). Moreover, the lipases (8) included in the predicted secretome could be one of the virulence factors, because lipases hydrolyze phospholipids, which are the main constituent of the plasma membrane [75].

Additionally, the secretomes of other blackleg fungal pathogens (*P. lingam* JN3 and *P. biglobosus*) were predicted for further comparison with *P. lingam* CAN1 (Table 2). The secretomes of *P. lingam* JN3 and *P. biglobosus* CA1 were retrieved from the NCBI database. The number of protein models in the *Plendomus* strains ranged from 11,837 to 12,469. A pipeline of programs was used to predict the secretome of each strain. The highest total number of predicted secretomes was recorded in *P. biglobosus* CA1, representing 1.95% of its total proteome. For the two *P. lingam* strains, the number of predicted proteins in the secretome of CAN1 is greater than that of JN3.

**Figure 6:**
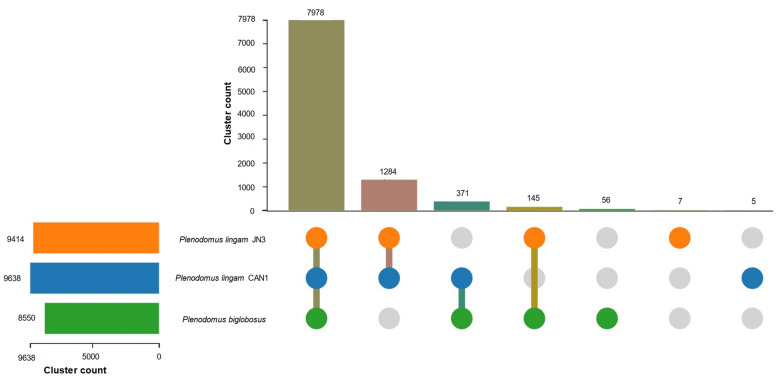
Orthologous clusters analysis between the three blackleg strains.

**Figure 7:**
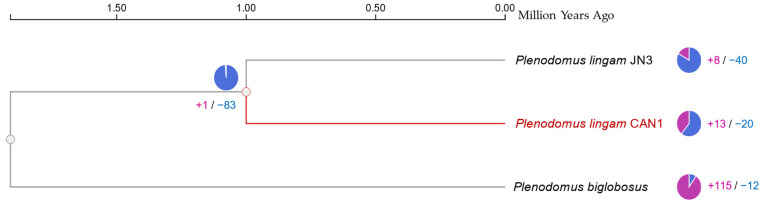
Phylogenetic tree, gene family contraction and expansion and timeline divergence (million years ago) of *Plenodomus* species. The pie charts show the expanded (purple) and contracted (blue) gene family proportions among all gene families.

For comparative genomic studies, orthologous clustering analysis was performed by OrthVenn3, which provides effective cluster identification for numerous species (Figure 6). Results revealed that the studied strains shared 7978 cluster genes with the strongest association between the two strains of *P. lingam* (1284 clusters), which was further confirmed by the overlapping cluster information in pairwise fashion among the three blackleg pathogens using heatmap analysis (Figure S1). A phylogenetic tree of the three pathogenic strains was designed (Figure 7). This ultrametric tree was constructed using protein sequences of three species, showing gene family expansion and contraction (Figure 7). The variation of gene family was calculated by CAFE5 [76]. *P. lingam* CAN1 and *P. lingam* JN3 were clustered into one branch. Differentiation time analysis showed that the two strains differentiated approximately one million years ago. This analysis illustrates the closer evolutionary relationship between *P. lingam* CAN1 and *P. biglobosus*, where *P. lingam* CAN1 is considered the intermediate strain between *P. lingam* JN3 and *P. biglobosus*.

**Figure 8:**
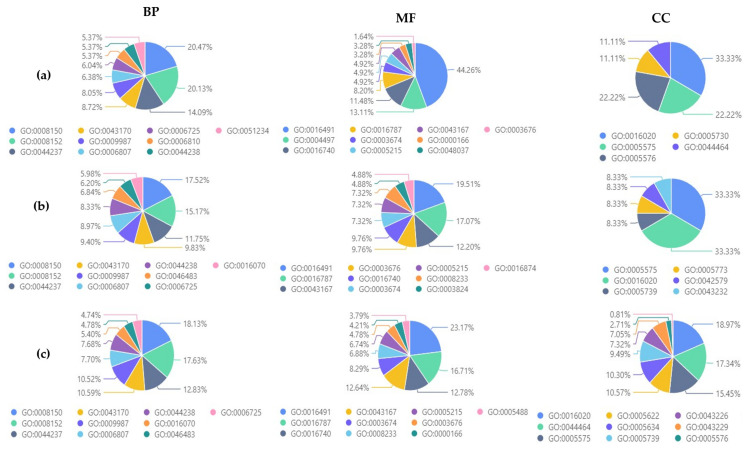
Biological process (BP), molecular function (MF), and cellular component (CC) of: (**a**) *P. lingam* CAN1 vs. *P. lingam* JN3; (**b**) *P. lingam* CAN1 vs. *P. biglobosus* CA1; (**c**) *P. lingam* CAN1 vs. *P. lingam* JN3 vs. *P. biglobosus* CA1.

**Figure 9:**
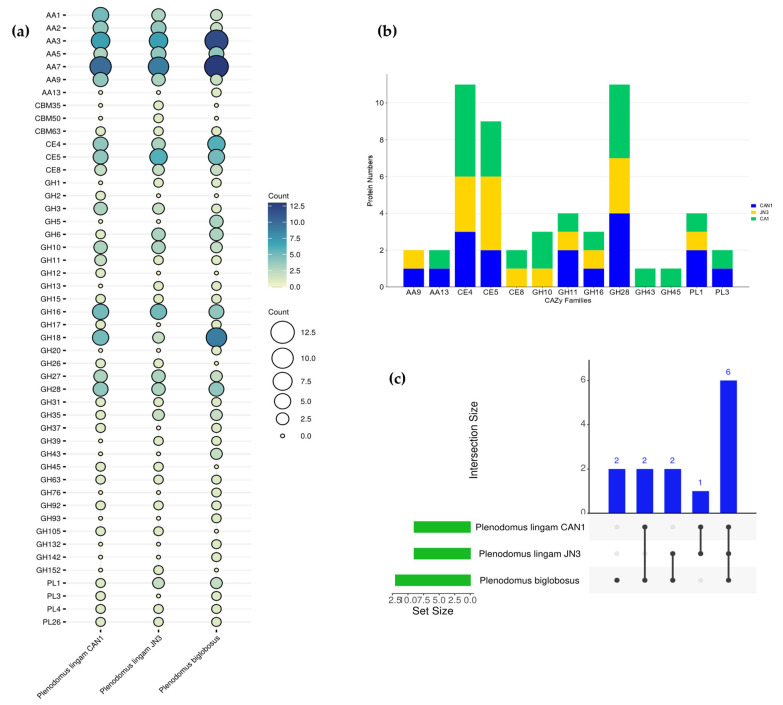
Comparison of carbohydrate-activating enzymes (CAZymes) between *Plenodomus* species: (**a**) Bubble plot showed different CAzymes in the secretome between the species; (**b**) Bar graph showed different CAZy families of effector proteins between species; (**c**) UpsetR plot showed number of CAZY families of effectors shared between species to each other.

When comparing the studied strains in terms of their gene ontology terms (Figure 8), it was found that the most shared biological processes were carbohydrate catabolic process, metabolic process, and cellular metabolic process, while oxidoreductase activity, monooxygenase activity, and transferase activity were the most identified molecular function. Also, the most common shared cellular components were membrane, cellular component, and extracellular region. The three studied strains showed a similar pattern of CAZymes (Figure 9): glucooligosaccharide oxidase (AA7), GMC oxidoreductase (AA3), chitin deacetylase (CE4), and acetyl xylan esterase (CE5) were the most identified CAZymes.

### 3.4. Analysis of Putative Effectors

Most effectors’ prediction approaches use structural characteristics of proteins and conserved domain (CD) of motifs. However, all effector proteins don’t share all these structural characteristics between species even if they share little sequence similarities. All these reasons make the prediction of effectors a challenging task [17]. We used a machine learning program (EffectorP) for building a model depending on a variety of features that predict cytoplasmic effectors (rich with positively charged amino acids) and apoplastic effectors (rich with cysteine residues) [46,77]. The entire predicted secretome of *P. lingam* CAN1 was screened for protein effectors, as illustrated in the pipeline scheme of this work (Figure 1). From the refined secretome, 49 effector proteins were described (Table 3). Seven candidates were found with both apoplastic and cytoplasmic effectors. Of the 49 putative effectors, the functional domain of 39 proteins was annotated. One putative effector (KAH9872288.1) possessed an unknown function domain (DUF6060), and the rest had no annotation. The absence of prediction to some sequences might be of interest for further analysis [78,79].

**Figure 10:**
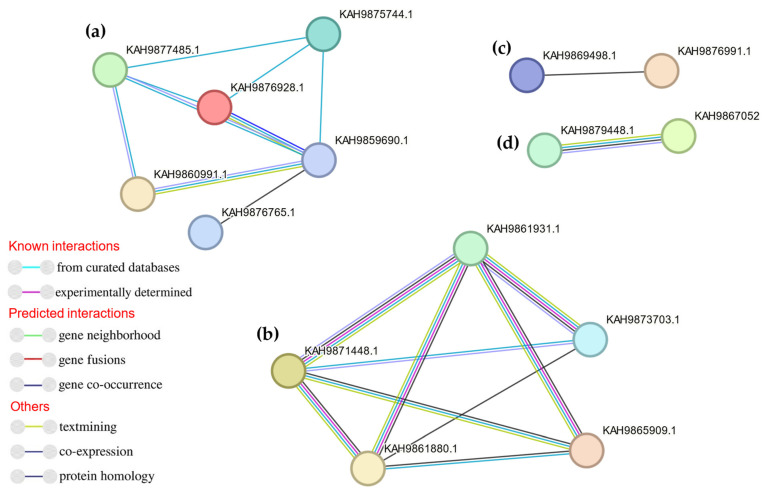
Protein–protein interactions between the associated effector proteins: (**a**) Interactions between pectate hydrolytic effector groups; (**b**) Interactions between protein disulphide isomerase effectors.; (**c**) Interactions between hemi-cellulase effector groups; (**d**) Interactions between peptidase S1 family effectors.

**Figure 11:**
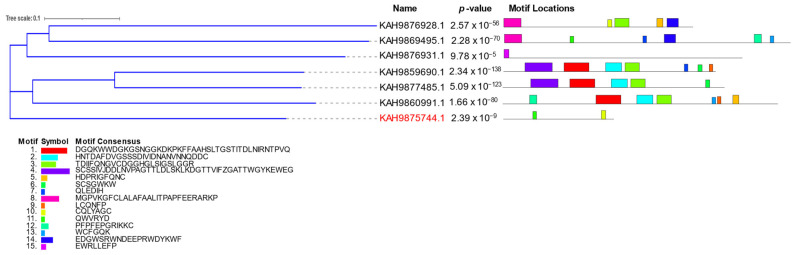
Constructed phylogenetic tree by Clustal Omega between pectin hydrolytic-related proteins of *P. lingam* CAN1 and analysis of motif locations by MEME tool.

The protein–protein interaction between putative effectors was analyzed by STRING V11.5 [48]. The dominant associated effector proteins were disulfide isomerase enzyme and pectin hydrolytic enzymes belonging to different families such as GH28, PL1, and PL2 (Figure 10). The other disconnected nodes were hidden from the network. Pectinolytic enzymes have a pivotal role in cell wall degradation and softening plant tissues [80]. The disulfide isomerase is an endoplasmic reticulum (ER) protein involved in protein folding and production of reactive oxygen species (ROS) that play a role in host–pathogen interaction [81]. Phylogenetic tree and MEME motifs were analyzed to CAZy families related to pectin degradation, as shown in Figure 7. Phylogenetic analysis illustrated that KAH9875744.1 (pectate lyase) was grouped into a single branch, although there are other proteins with the same domain such as KAH9859690.1 and KAH9860991.1. M1–M15 motifs of pectinolytic-related proteins were compared. KAH9859690.1 and KAH9859690.1 had more similar motifs, while KAH9876931.1 and KAH9875744.1 showed obviously different motifs (Figure 11). From this comparison, we discovered that M15 was found only in KAH9876931.1 protein and M10 specifically presented in KAH9875744.1 and KAH9876928.1. According to phylogenetic relationships, KAH9875744.1 was grouped singly despite protein–protein interaction results and similarity with other protein domains. The results of MEME analysis were mostly inconsistent with those of the phylogenetic analysis, except in the middle clade. These findings are different from previous work [82,83].

**Figure 12:**
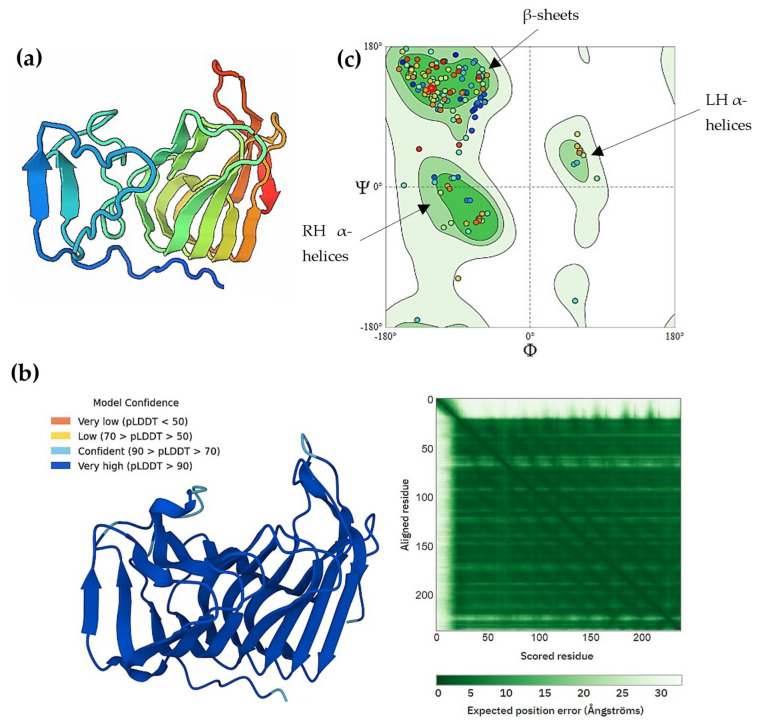
Pectate lyase 3D homology structure and structure assessments: (**a**) Predicted 3D homology model structure of pectate lyase via SWISS-MODEL server; (**b**) 3D modeled structure prediction and expected prediction error using AlphaFold2 database; (**c**) Ramachandran plot of the model structure validation shows favorable β-sheet and α-helix angles.

**Figure 13:**
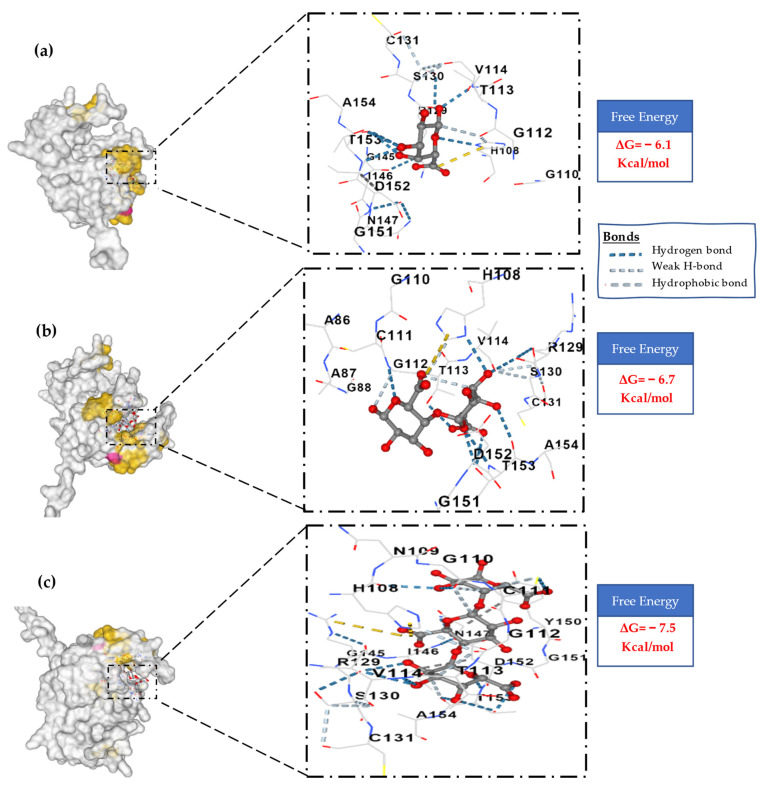
Molecular docking modeling between pectate lyase (protein) and (**a**) Pectin (ligand); (**b**) Digalacturonate (ligand); (**c**) Pectate (ligand).

For more confirmation, structure protein prediction (homology modeling) and molecular docking were investigated for the KAH9875744.1 protein by SWISS-MODEL and CB-Dock2, respectively. The best model match with the candidate protein KAH9875744.1 was pectate lyase template of *Colletotrichum nymphaeae* SA-01 with GMQE equal to 0.93, 70.59% sequence identity, and 87.57% Ramachandran favored, all indicating the good quality of the modeling protein (Figure 12a–c). Also, the same result was obtained when AlphaFold was applied [84] to the protein database and gave pLDDT about 98% confidence (very high model confidence, Figure 12b). These results confirm that the KAH9875744.1 protein is pectate lyase, where the 3D protein structure is highly related to its function; they also predict the binding sites of the protein [85]. The downloaded 3D structure model (pectate lyase) was used for docking analysis against three ligands (pectin, digalacturonate, and pectate). The estimated free energy of binding between the protein and pectin is −6.1 Kcal/mol (Figure 13a) and the free energy between the protein and digalacturonate is −6.17 Kcal/mol (Figure 13b), while it is estimated about −7.5 Kcal/mol between the protein and pectic acid (Figure 13c). Also, pectate forms a stronger hydrogen bond than other ligands for binding to the receptor protein. These results indicate that pectate is a good ligand for the 3D model protein, which confirms the previous findings about the KAH9875744.1 protein.

In comparison to effector proteins secreted by other related blackleg fungi, *P. lingam* JN3 and *P. biglobosus* secrete 42 and 63 effectors, respectively (Table 2, Tables S3 and S4). CAZyme classifications for *P. lingam* JN3 and *P. biglobosus* were annotated (Tables S5 and S6). The three *Plenodomus* species shared six CAZy enzyme families: CE4, CE5, GH11, GH16, GH28, and PL1 (Figure 9b,c). *P. lingam* CAN1 and *P. lingam* JN3 shared only the AA9 family, while two CAZy families (AA13 and PL3) were secreted from both *P. lingam* CAN1 and *P*. *biglobosus* (Figure 9b,c). Orthologue analysis found that *P. lingam* CAN1 shared 14 CAZy effectors with other related species. The comparison of putative effectors and CAZy families between the three pathogenic fungi ensures the role of pectinolytic and hemicellulolytic enzymes for starting the pathogenesis process against the rapeseed plants.

### 3.5. Analysis of Virulence Factors

The Pathogen-Host Interaction database (PHI base) was used to compare and blast the putative effector proteins with pathogenicity genes homologous to other phytopathogens [86]. These data classify the proteins into different categories, such as reduced virulence, unaffected pathogenicity, hypervirulence, loss of pathogenicity, and effector (plant avirulent determinant), among others [87]. Based on the PHI annotation, most effector proteins were classified as reduced virulence and unaffected pathogenicity (Table 3), meaning that the transgenic strain which expresses no, or reduced, levels of a specific gene product has wild-type disease. These findings agreed with Urban et al. [47], who classified 44% of data entries as phenotype term ‘reduced virulence’, followed by the unaffected pathogenicity term (26%). Two effectors (KAH9867270.1 and KAH9865263.1) were classified as hypervirulence (increased virulence). Both proteins are homologous to the same organism and MfCUT1 gene in *Monilia fruticola* (Table S7). This gene is a redox-regulated cutinase gene that increases the virulence of the brown rot caused by the fungus [88]. Four proteins were assigned as effector (plant avirulence determinant), including two genes (Vd5LysM and GIP) [89]. The KAH9861890.1 protein (Ubiquitin3-bd_dom) was encoded by the NPS6 gene that is involved in siderophore-mediated iron metabolism (conserved virulence determinant of plant pathogenic ascomycetes) [90]. The three effectors containing a chitin-binding domain (KAH9880554.1, KAH9877596.1, and KAH9865021.1) might be of interest because the interactions of protein effectors with chitin to suppress the chitin-induced defence response is a mechanism used by known effectors such as Avr4 or Ecp6 [91]. Surprisingly, these proteins were encoded by the CDA2 (cytidine deaminase) gene, and none of these contain a LysM domain which is found in the Ecp6, so these proteins were classified as unaffected pathogenicity. Also, two effectors (KAH9879448.1 and KAH9867052.1) were found by a BLAST against the PHI database as an effector (plant avirulence determinant) that were encoded by GIP1 (PHI:652) and GIP2 (PHI:653) of *Phytophthora sojae* (Table S7). These proteins are serine endopeptidases acting as inhibitors of the endo-β-1,3-glucanases of the plant host to suppress its elicitor-mediated defence response [92]. Four proteins were matched with protein disulphide isomerase *pdi* genes (PHI:9867) of *Ustilago maydis*. Because *P. lingam* has a hemibiotrophic lifestyle, such cell-death-inducing genes might have a substantial role during the necrotrophic stage of the pathogen.

## 4. Conclusions

This work provides the first in silico exploration of *P. lingam* CAN1 global secretome and compares it with other related species (*P. lingam* JN3 and *P. biglobosus*). To shed light on the infection mechanisms of *P. lingam* CAN1 on *Brassica napus*, we annotated the first draft genome of the pathogenic fungus and analyzed its secretory proteins, including putative effectors and virulence factors. Out of the refined secretome (217 proteins), 85 CAZymes, 25 proteases, 8 lipases, and 49 putative effector proteins were characterized. Varieties of effector virulence factors were detected when matched to the PHI-base database. Most of them were categorized as reduced and unaffected pathogenicity, while two effectors were classified as hypervirulence. The effector proteins include all cell-wall-degrading enzymes, especially pectinolytic and hemicellulolytic enzymes where the degradation of xylan and pectin is required for penetrating and proliferating the pathogen inside host cells. Also, the comparative genetic analysis between *P. lingam* CAN1 and other closely related strains shared most of the CAZYmes and effectors. The present study will be a worthy source for studies related to understating the pathogenicity mechanisms between pathogenic *Plenodomus* species and their plant hosts.

## Figures and Tables

**Table 1:** List of CAZymes in the secretome of *Plenodomus lingam* CAN1.

CAZy Family	Annotation	InterPro ID	EC Number	Substrate	Copy No.
AA1	Multicopper oxidase	IPR045087	1.10.3.2	Lignin	5
AA2	Lignin peroxidase	IPR001621	na	Lignin	4
AA3	GMC oxidoreductase	IPR012132	1.1.99.18	Cellobiose	7
AA5	Radical Copper oxidase	-	1.3.3.9	Galactose	3
AA7	Glucooligosaccharide oxidase	-	1.3.3.-	Cellobiose	10
AA9	lytic cellulose monooxygenase	IPR005103	1.14.99.56	Cellulose	4
CBM63	Cellulose binding	IPR007112	na	Cellulose	1
CE4	chitin deacetylase	-	3.5.1.41	Chitin	4
CE5	acetyl xylan esterase	IPR000675	3.1.1.72	Xylan	4
	Cutinase	IPR011150	3.1.1.74	Cutin	
CE8	Pectin methylesterase	-	3.1.1.11	Pectin	2
GH2	β -mannosidase	-	3.2.1.25	Mannose	1
GH3	β-glucosidase	IPR017736	3.2.1.21	Cellulose	3
GH6	1, 4- β-cellobiohydrolase	IPR016288	3.2.1.91	Cellulose	1
GH10	Endo-β-1,4-xylanase	IPR044846	3.2.1.8	Xylan	3
GH11	Endo-β-1,4-xylanase	IPR001137	3.2.1.8	Xylan	2
GH12	Endo-β-1,4-glucanase	IPR002594	3.2.1.4	Cellulose	1
	Xyloglucanase	-	3.2.1.151	Xylan	
GH15	Glucoamylase	IPR000165	3.2.1.3	Starch	1
GH16	Endo-β-1,3-galactanase	IPR000757	3.2.1.181	Galactan	5
GH17	Endo-1,3-β-glucosidase	IPR017853	na	Polysaccharides	1
GH18	Chitinase	IPR001223	3.2.1.14	Chitin	5
GH26	Endo-β-1,4-glucanase	IPR000805	3.2.1.4	Cellulose	1
GH27	α-galactosidase	IPR002241	3.2.1.22	Hemicellulose	3
GH28	Polygalacturonase	IPR000743	3.2.1.15	Pectin	4
GH31	α -glucosidase	IPR000322	3.2.1.20	Amylose	1
GH35	β-galactosidase	IPR001944	3.2.1.23	Hemicellulose	1
GH37	α, α-trehalase	IPR001661	3.2.1.28	Trehalose	1
GH45	Endo-β-1,4-glucanase	-	3.2.1.4	Cellulose	1
GH63	α-glucosidase	IPR004888	3.2.1.106	Oligosaccharides	1
GH92	α-mannosidases	IPR044846	na	Mannose	1
GH105	Rhamnogalacturonyl hydrolase	IPR010905	3.2.1.172	Pectin	1
PL1	Pectate lyase	-	4.2.2.2	Pectin	1
PL3	Pectate lyase	IPR004898	4.2.2.2	Pectin	1
PL4	Rhamnogalacturonan lyase	IPR029413	4.2.2.-	Galacturonan	1
PL26	Rhamnogalacturonan exolyase	-	4.2.2.24	Galacturonan	1

**Table 2:** Comparison of secretome data between the three blackleg fungal strains.

Fungal Species	Total Proteome	Secretome (%)	CAZymes	Effectors
*P. lingam* CAN1	11,837	217 (1.83%)	85	49
*P. lingam* JN3	12,469	209 (1.67%)	90	42
*P. biglobosus* CA1	12,183	238 (1.95%)	103	63

**Table 3:** Characterization of putative effectors in the refined secretome of *P. lingam* CAN1.

Protein ID	Effector Probabilities		PHI-Blast		Domain
	Apoplastic	Cytoplasmic	PHI ID	Classification	
KAH9881423.1	0.646	-	PHI:10423	Reduced virulence	-
KAH9881503.1	0.658	-	PHI:10461	Reduced virulence	-
KAH9880351.1	-	0.844	PHI:5599	Unaffected pathogenicity	-
KAH9880478.1	-	0.549	PHI:9191	Reduced virulence	SET_dom
KAH9880542.1	0.677	-	PHI:2138	Unaffected pathogenicity	Cellulose/chitin-bd_N
KAH9880554.1	0.78	-	PHI:6391	Unaffected pathogenicity	Chitin-bd_1/NODB_dom
KAH9879448.1	0.826	-	PHI:652	Avirulence determinant	Trypsin_dom
KAH9876765.1	0.504	-	PHI:6228	Reduced virulence	GH16_dom
KAH9876928.1	-	0.666	PHI:1028	Reduced virulence	Pectinesterase_cat
KAH9876931.1	0.627		PHI:1034	Unaffected pathogenicity	Pectin_lyase_fold
KAH9876991.1	-	0.791	PHI:9553	Unaffected pathogenicity	ML_PG-PI_TP
KAH9877485.1	0.698	-	PHI:2817	Unaffected pathogenicity	Glyco_hydro_28
KAH9877596.1	0.652	-	PHI:6391	Unaffected pathogenicity	Chitin-bd_1/NODB_dom
KAH9874320.1	0.861	0.527	PHI:8520	Reduced virulence	AltA1
KAH9874600.1	0.883	-	PHI:571	Unaffected pathogenicity	GH11_dom
KAH9875744.1	0.835	0.631	PHI:180	Reduced virulence	Pectin_lyas_fold
KAH9875764.1	0.723	-	PHI:2044	Loss of pathogenicity	Lipocln_cytosolic-bd
KAH9875892.1	-	0.525	PHI:10926	Reduced virulence	-
KAH9873589.1	0.793	-	PHI:6832	Avirulence determinant	LysM_dom
KAH9873703.1	-	0.619	PHI:9867	Reduced virulence	Thioredoxin
KAH9873874.1	-	0.544	PHI:10459	Reduced virulence	MRH_dom
KAH9874050.1	0.826	-	PHI:3214	Unaffected pathogenicity	CFEM_dom
KAH9872236.1	0.518	-	PHI:9546	Reduced virulence	Aspergillopepsin-like
KAH9872288.1	0.758	-	-	-	DUF6060
KAH9870266.1	0.525	-	PHI:6823	Reduced virulence	-
KAH9871448.1	-	0.698	PHI:9867	Reduced virulence	ERp29_C/Thioredoxin
KAH9869412.1	-	0.863	PHI:3440	Reduced virulence	-
KAH9869498.1	-	0.532	PHI:9903	Reduced virulence	PCSK9_ProteinaseK
KAH9869606.1	0.794	0.56	PHI:8208	Reduced virulence	-
KAH9867862.1	-	0.659	PHI:401	Unaffected pathogenicity	-
KAH9868053.1	-	0.703	PHI:1380	Unaffected pathogenicity	-
KAH9868081.1	0.504	-	PHI:2210	Reduced virulence	GH11_dom
KAH9867052.1	0.693	-	PHI:653	Avirulence dominant	Trypsin
KAH9867270.1	0.723	-	PHI:2383	Hypervirulence	Cutinase_monf
KAH9867337.1	0.713	0.79	PHI:6834	Avirulence dominant	LysM
KAH9864751.1	0.527	-	PHI:197	Reduced virulence	Oxid_FAD_bind_N
KAH9865909.1	-	0.676	PHI:10459	Reduced virulence	MRH_dom/PRKCSH_N
KAH9864831.1	-	0.639	PHI:4231	Reduced virulence	Fasciclin (FAS1)
KAH9865021.1	0.844	0.649	PHI:3972	Unaffected pathogenicity	Chitin-bd_1
KAH9865263.1	0.78	0.602	PHI:2383	Hypervirulence	Cutinase/axe
KAH9861880.1	-	0.757	PHI:5236	Reduced virulence	DnaJ_dom
KAH9861890.1	0.777	-	PHI:1008	Reduced virulence	Ubiquitin3-bd_dom
KAH9861931.1	-	0.578	PHI:9867	Reduced virulence	Thioredoxin
KAH9862482.1	-	0.575	PHI:181	Reduced virulence	Znf_C2H2_type
KAH9860991.1	0.562	-	PHI:3503	Reduced virulence	Pectin_lyas_fold
KAH9861149.1	0.505	-	PHI:1087	Unaffected pathogenicity	Tyrosinase_Cu-bd
KAH9861329.1	0.816	-	PHI:11528	Reduced virulence	NODB_dom
KAH9862149.1	0.501	-	PHI:10358	Reduced virulence	Cellulose-bd_dom_fun
KAH9859690.1	0.698	-	PHI:7283	Reduced virulence	Pectin_lyas_fold

## Data Availability

Not applicable.

## Supplementary Materials

The following supporting information can be downloaded at: https://www.mdpi.com/article/10.3390/jof9070740/s1, Figure S1: Pairwise heatmap showing the relationship between the species after the clustering orthologue analysis; Table S1: List of the proteins that represent the proteome of *P. lingam* CAN1; Table S2: Physicochemical characters of *P. lingam* CAN1 refined secretome; Table S3: Effector proteins secreted from *P. lingam* JN3 that were detected by EffectorP; Table S4: Effector proteins secreted from *P. biglobosus* CA1 that were detected by EffectorP; Table S5: CAZy families of *P. lingam* JN3 secreted proteins that were annotated by dbCAN3; Table S6: CAZy families of *P. biglobosus* CA1 secreted proteins that were annotated by dbCAN3; Table S7: Virulence factor genes with matched organisms against *P. lingam* CAN1 using PHI database.
