# Supplementary material for: Secretome Analysis for a New Strain of the Blackleg Fungus Plenodomus lingam Reveals Candidate Proteins for Effectors and Virulence Factors
Source: J Fungi (Basel). 2023 Jul 11;9(7):740. doi: 10.3390/jof9070740 (PMC10381368; doi:10.3390/jof9070740)
Supplement: Supplementary file 1 [file jof-09-00740-s001.zip › Figure S1.pdf]

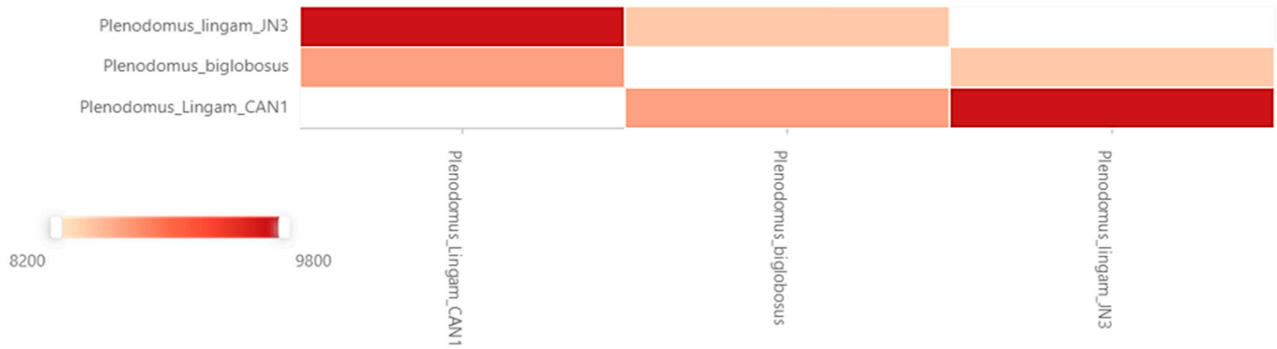

Figure S1: Pairwise heatmap showing the relationship between the species after the clustering orthologue analysis.
